# Arabic translation and psychometric testing of the prenatal eating behaviors screening tool

**DOI:** 10.1186/s40337-025-01264-4

**Published:** 2025-07-15

**Authors:** John Paul Ben Silang, Fathima Minisha, Hayat Qassim A-Hajjaji, Catherine Medina Lim, Jishamol Thankappan, B’Chira Bent Ismail Ben Ali, Mani Megalai Prakash, Zeinab Abdillahi Mohamed, Remya Mathew, David Hali De Jesus, Salwa Mohammad Abu Yaqoub, Thomas Farrell

**Affiliations:** 1 Department of Research, Women’s Wellness and Research Center, Hamad Medical Corporation, Doha, Qatar; 2 Nursing Department, Women’s Wellness and Research Center, Hamad Medical Corporation, Doha, Qatar; 3 Obstetrics and Gynecology Department, Women’s Wellness and Research Center, Hamad Medical Corporation, Doha, Qatar; 4 College of Medicine, Qatar University, Doha, Qatar

**Keywords:** Eating behaviors, Eating disorders, Prenatal eating disorders screening, PEBS, Psychometric analysis

## Abstract

**Background:**

Early detection and treatment of eating behavioral problems among pregnant women are essential due to the associated adverse impact on pregnancy and the health of the offspring. Prenatal Eating Behaviors Screening (PEBS) tool, a 12-item self-administered questionnaire, can be used to screen for eating disorders (ED) during pregnancy. This study performed an Arabic translation of PEBS and a psychometric analysis to determine its validity and reliability.

**Methods:**

The standard forward-backward translation method was used to generate PEBS-Arabic. The 12-item Likert scale questionnaire was completed by 116 antenatal women in the tertiary maternity care hospital in Qatar. Content validity was determined by the content validity index (CVI) using input from five experts. Reliability was assessed using Cronbach’s alpha, and confirmatory and exploratory factor analysis (CFA & EFA) to test construct validity. The correlations between PEBS-Arabic scores and maternal characteristics were explored.

**Results:**

The mean total PEBS score in the cohort was 16.3 (± 5.2), with nulliparity and higher educational level resulting in statistically significantly higher mean scores. The PEBS-Arabic had a very good item-CVI and scale-CVI of 1.00. The overall Cronbach’s alpha was 0.77, which demonstrated good and acceptable reliability. The CFA using a single-factor solution showed an acceptable correlation for most items. In the EFA, a two-factor solution resulted in most items loading accurately into the pre-determined factors (bulimia and anorexia) with acceptable correlations.

**Conclusion:**

The PEBS-Arabic is the first translated version of this pregnancy-specific screening tool for ED. This tool demonstrates good reliability, content and construct validity. This study is a valuable step towards understanding and detecting the prevalence and determinants of ED in pregnancy, with the aim of improving maternal, fetal and child health.

## Abstract (plain language summary)

The study aimed to determine the validity and reliability of the Prenatal Eating Behaviors Screening (PEBS) tool, a 12-item self-administered questionnaire, for early detection and treatment of eating disorders during pregnancy. The Arabic translation of PEBS and psychometric evaluation was performed. Moreover, a survey was conducted using PEBS which followed a six-point Likert scale which were completed by 116 antenatal women in Qatar. The results showed good reliability, and content and construct validity. The PEBS-Arabic is the first translated version from original English. This study is crucial for understanding and detecting the prevalence and determinants of eating disorders in pregnancy, ultimately improving maternal, fetal, and child health.

## Background

A balanced diet when pregnant is vital for the development and lifelong health of the offspring [1]. A balanced nutritional status may prevent congenital malformations, premature birth, and low birth weight [2, 3]. During prenatal or antenatal care, nutritional education opportunities and health promotion tools can help expecting parents overcome several challenges in pregnancy, including maladaptive eating patterns or behaviors [1].

Many genetic, psychological and social factors affect eating behaviors, influencing meal timings and the type and amount of food consumed [4]. Disruptions in eating habits like frequent restriction, skipping meals, eating beyond fullness, and continuous thoughts about body weight may lead to eating disorders (ED) [5], with the prevalence in pregnancy ranging from 7% to nearly 20% [6–8]. Women with eating disorders exhibit lower cortisol levels than those with a balanced diet, indicating a potential dysfunction in hypothalamic-pituitary function. This may lead to an increased stress response in infants and pose a risk for future psychological and physical health issues. [9] Previous studies about eating disorders showed that anorexia nervosa, defined as severe restriction from food due to fear of gaining weight, was associated with fetal growth restriction, anomalies and preterm birth [10]. Also, binge-eating, defined as consuming excessive food without purging, was associated with increased weight gain and large-for-gestational-age babies [10]. The benefits of early detection and treatment of eating disorders make screening methods, such as the use of survey instruments, crucial to provide timely therapy.

The current challenge is appropriately identifying abnormal eating behaviors in antenatal women. They are often not revealed as women are concerned about the associated social stigma or due to a lack of enquiry about symptoms by the healthcare workers [11]. A review study showed that self-reported questionnaires are, therefore, more likely to reveal EDs, such as the Eating Disorder Examination Questionnaire (EDE-Q), the Eating Disorder Inventory-2 (EDI-II), the Disordered Eating Behavior Scale (DEBS) and the SCOFF questionnaire used to screen for ED in the general population [12] A recent systematic review showed that although DEBS, EDE-Q and EDI-II may be valid and reliable when applied in pregnancy, they have unclear specificity as often symptoms of ED overlap with physiological changes in pregnancy. The validations tests are primarily restricted to internal consistency, which limits their suitability in detecting eating disorders during pregnancy [12]. Instruments such as SCOFF and EDE have also been adapted to screen the pregnant population for eating disorders [12, 13]. However, SCOFF has poor internal consistency and was unable to accurately capture ED in pregnancy, while EDE, although having good reliability, was evaluated only in pregnant women with higher body mass index. Hence, Claydon et al. developed the Prenatal Eating Behaviors Screening (PEBS) tool to address these gaps. The PEBS was developed in English and was reviewed and tested, which confirmed good reliability (internal consistency or α = 0.96) and sensitivity (86.5%) in screening for ED in pregnancy [14].

A significant gap in the current literature exploring the eating behaviors of pregnant women is the deficiency of studies from countries in the Middle East, such as Qatar. Studies from Lebanon report psychometric properties of the Arabic translation of the Disordered Eating Attitudes in Pregnancy Scale (DEAPS), to detect body image perceptions and attitudes towards eating in pregnancy rather than as a screening tool [15]. PEBS is a reliable and valid tool with no previously validated Arabic translations. A translated version is useful within the Arab context, using terms that can be understood by most Arabic-speaking women delivering in the country. Cultural differences potentially exist in the perception and attitudes towards eating behaviors, as shown in previous studies done in the Middle East. Therefore, translating and validating PEBS becomes necessary for early detection and providing culturally appropriate perinatal interventions. In this study, we translated the PEBS tool into Modern Standard Arabic (MSA) and performed psychometric testing including content validity, reliability and confirmatory factor analyses. Furthermore, the correlations between socio-demographic profiles among native Arabic-speaking pregnant women and eating behavior scores were explored.

## Methods

### Setting and participants

The Arabic translation of the PEBS self-administered questionnaire was performed in Women’s Wellness and Research Centre (WWRC), a tertiary maternity facility in Doha, Qatar. The translated version, PEBS-Arabic, was administered to native Arabic-speaking pregnant women in their third trimesters (> 28 weeks gestational age), admitted to the antenatal units. Through convenience sampling, prenatal mothers who were available and accessible in these units were recruited, which ensured a high response rate of 98%. The inclusion criteria included maternal age between 18 and 40 years and the ability to comprehend, read and write the Arabic language. Women who have answered the questionnaire any time previously, of other non-Arab nationalities regardless of Arabic language proficiency, and those having high-risk pregnancies were excluded. Considering that PEBS contains 12 items, the sample size required for testing reliability was set at 120 participants. Since this was 10 times the number of variables, it was considered adequate for appropriate factor analysis as well.

### Instrumentation

The Prenatal Eating Behavior Screening or PEBS tool was developed by Claydon et al. in 2022 [14] to identify potential eating disorders across pregnancy trimesters and ensure early detection and treatment. The PEBS, initially written in English, consists of 12 items. The items are scored from 1 to 5 on a Likert scale:"1"indicates never or strongly disagree, and"5"indicates either daily or strongly agree. The total scores range from 12 to 60. The items in PEBS correlate more with certain ED diagnoses such as bulimia, binge eating (BE) and anorexia. Items 1–4, 6 and 8 help screen for bulimia, 7–9 for BE, and 5 and 9–12 for anorexia. A total score ≥ 39 is considered a higher risk for ED, as defined by the authors. The first page includes information regarding participant demographics such as age, marital status, education, number of previous pregnancies and children, employment, past diagnosis of ED, type and treatment sought and weight change during pregnancy.

### Translation and adaptation of the instrument

Following the World Health Organization’s guidelines relevant to scales in population health research cited in various studies [16], we used the forward–backward method for translation. In forward translation, two clinicians in Obstetrics and Gynecology, proficient in both English and Arabic, translated the tool into PEBS-Arabic. Another bilingual clinician, unfamiliar with the English version, performed the backward translation by translating the PEBS-Arabic back to English. Reconciliation followed, and a cognitive review was done with three eligible participants from the target population who were asked to answer the revised tool, and their consensus decision was to proceed with adapting the tool for psychometric evaluation. Judgement to clinical relevance and adaptation of each item of the tool was done from this stage up to validity and reliability testing.

### Ethical considerations

The Institutional Review Board of the Medical Research Center, Hamad Medical Corporation, approved the conduct of the study (MRC 01–23–453). Permissions were sought from the authors of the original PEBS tool for adaptation, translation and psychometric testing. Detailed information about the study was provided to participants using research information sheets in order to obtain voluntary participation prior to data collection.

### Data analysis

#### Content validity

We followed the recommended guidelines for evaluating the content validity [17] using the content validity index, both individual items (I-CVI) and overall scale (S-CVI). Five experts reviewed and scored the items according to degree of relevance from 1 (not relevant) to 4 (highly relevant), based on their opinion regarding the usefulness of each item towards defining the main construct. The scale was made binary by combining scores 1 and 2 as “not relevant” and 3 and 4 as “relevant”. The I-CVI was computed as the number of experts rating “relevant” divided by the total number of experts, for each individual item. Since five experts participated, the I-CVI was expected to be 1.00 as per recommendations. The S-CVI was calculated as the proportion of items that achieved a similar rating of 3 or 4 by all the content experts and was expected to be ≥ 0.9.

#### Construct validity

Confirmatory factor analysis (CFA) was performed using a polychromic matrix with varimax rotation using the recommended single-factor solution [14]. Factor loadings of more than 0.65 were considered acceptable. The variation explained by the factor was determined, and the fit of the model was expressed using AIC (Akaike Information Criteria). An exploratory factor analysis was performed using two-factor and three-factor solutions since the items are intended to screen for distinct conditions such as bulimia, BE and anorexia.

#### Reliability

For the assessment of reliability, Cronbach's alpha was calculated, including all 12 items and the inter-item correlations. An alpha of more than 0.7 [18] and an inter-item correlation of 0.2–0.5 was considered good and acceptable reliability or internal consistency.

#### PEBS-Arabic scores and correlation with demographics

The total score was calculated as the sum of each participant's individual item scores. The mean total score was represented as mean ± standard deviation (SD) and median and interquartile range (IQR). The mean/median total scores were calculated according to maternal demographic categories, and comparisons were made between groups using the Wilcoxon rank-sum test since each item represents an ordinal scale. The proportion of women choosing each option and the mean scores for each item were also calculated and represented in bar graphs. All statistical analysis was performed using Stata Statistical Software, Release 18, College Station, TX: StataCorp LLC [19].

## Results

A total of 116 women completed the questionnaire. No concerns were raised by the participants regarding comprehension of the items in PEBS-Arabic. The mean total score in the cohort was 16.3 (SD = 5.2, median = 14, IQR-12–18.5). Table 1 shows the mean scores in the different maternal demographic categories. More than 18% of the women had a postgraduate or master's education and had significantly higher mean scores than the 31% who completed secondary school education only (18, IQR = 12–24 versus 13, IQR = 12–15, score difference = 5.0, *p* = 0.011). Similarly, nulliparous women (i.e. women who had never given birth) (33%) had much higher scores compared to grand multiparous women (7%) (18, IQR = 13–24 versus 12, IQR = 12–14.5; score difference = 6.0, *p* = 0.001).

**Table 1:** Participant demographics and PEBS total scores

Participants total N = 116		Mean ± SD n (%N)	PEBS total scores		
			Mean ± SD	Median (IQR)	*P* value
Total Cohort		N = 116	16.3 ± 5.2	14 (12–18.5)	
Age in completed years		30.4 ± 4.9			
Age (Missing 2)	20–30 years	57 (50%)	16.8 ± 5.7	15 (12–19)	0.322
	31–40 years	57 (50%)	15.8 ± 4.6	14 (12–18)	
Marital status	Married	113 (97.4%)	16.4 ± 5.2	14 (12–19)	0.644
	Separated/Divorced/Widowed	3 (2.6%)	14.3 ± 3.2	13 (12–18)	
Education level (Missing 2)	School	35 (30.7%)	13.9 ± 2.5	13 (12–15)	0.011
	College	58 (50.9%)	16.8 ± 5.0	15.5 (12–19)	
	Postgrad/Masters	21 (18.4%)	18.9 ± 6.9	18 (12–24)	
Employment (Missing = 3)	Employed	55 (48.7%)	15.6 ± 4.7	13 (12–18)	0.216
	Unemployed	58 (51.3%)	16.9 ± 5.6	15 (12–19)	
Number of children		1.5 ± 1.6			
Number of children (Missing = 2)	0	37 (32.5%)	18.8 ± 6.0	18 (13–24)	0.001*
	1–3	69 (60.5%)	15.2 ± 4.3	13 (12–17)	
	≥ 4	8 (7.0%)	13.5 ± 2.6	12 (12–14.5)	
Number of previous pregnancies		2.4 ± 1.6			
Number of previous pregnancies (Missing = 2)	0	6 (5.3%)	16.5 ± 4.7	16 (12–19)	0.107
	1–3	83 (72.8%)	16.7 ± 5.3	15 (12–19)	
	≥ 4	25 (21.9%)	14.8 ± 4.5	12 (12–16)	
Diagnosed with ED previously (Missing = 7)	Yes	8 (7.3%)	17.4 ± 6.1	16 (12–22)	0.682
	No	101 (92.7%)	16.2 ± 5.2	14 (12–18)	
Weight change during pregnancy (Skipped question- 58)	Lost ≥ 15 pounds	7 (12.1%)	14.9 ± 2.9	14 (12–18)	0.486
	Lost < 15 pounds	2 (3.5%)	18.0 ± 8.5	18 (12–24)	
	Stayed the same	6 (10.3%)	17.0 ± 6.6	13.5 (12–25)	
	Gained < 15 pounds	13 (22.4%)	15.5 ± 5.0	13 (12–16)	
	Gained ≥ 15 pounds	30 (51.7%)	18.2 ± 6.5	16 (13–20)	

SD- standard deviation; IQR- interquartile range; ED- eating disorders; %- proportion of total after excluding missing data; Total score represents sum total of 12 individual item scores (each item score ranging from 1–5, min total = 12, max total = 60); significance testing done using Wilcoxon ranksum test, p < 0.05 considered strong evidence against null hypothesis

The women ranged in age from 20 to 40 years (mean = 30.4 ± 4.9), with younger women having slightly higher mean scores than the older. Similarly, married women and those unemployed had higher scores than their counterparts, although these differences in marital (*p* = 0.644) and employment (*p* = 0.216) were not statistically significant. The number of past pregnancies followed the same pattern as parity, and women with four or more pregnancies had lower scores. The mean scores were also higher in those with a previous ED diagnosis. However, the differences in the scores between the categories of previous pregnancies and ED diagnosis did not reach statistical significance. The question regarding weight change in pregnancy was skipped by 50% of women, and there were no statistically significant differences in scores between the categories. There were no statistically significant differences in the demographics of those who skipped this question and those who answered.

**Fig. 1:**
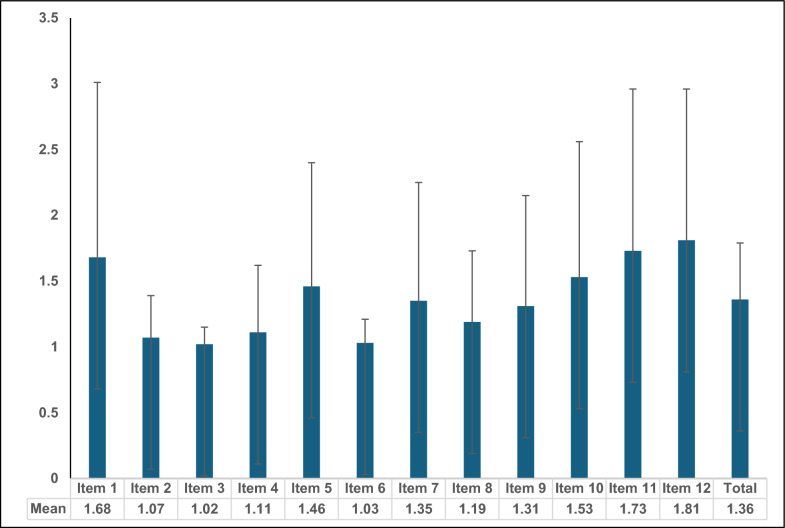
Mean scores for each item of the PEBS questionnaire; The error bars represent standard deviations Minimum score = 1, Maximum score = 5; Total represents the overall mean score per item of the questionnaire

The overall per-item score in the cohort was 1.36 out of 5. The mean scores for each item in PEBS-Arabic are shown in Fig. 1 and Table 2. Item 12 *(“During this pregnancy, how strongly do you agree that…you have had the desire for your stomach to feel hungry.”)* had the highest mean score of 1.8, followed closely by Items 11 and 1 (mean score = 1.7). Item 1 *(Have you used any pregnancy symptoms to control weight?)* had the highest proportion of women selecting the maximum score of 5 (daily symptoms- in 10%). More than 90% chose"Never"for Items 2, 3, 4, and 6, which are meant to screen for bulimia. Overall, the scores were higher for items related to anorexia (Items 5, 9, 10, 11, 12) compared to BE or bulimia in our population (Fig. 1).

**Table 2:** Distribution of PEBS scores for 12 items, with mean scores and testing for internal consistency (reliability)

ED content	PEBS items	PEBS Likert scale (n, %N)					Mean score	Item-test correlation	Item-rest correlation	Average inter-item correlation	Cronbach’s alpha
		Never (Score 1)	Rarely (Score 2)	Occasionally (Score 3)	Weekly (Score 4)	Daily (Score 5)					
Bulimia	Item 1	87 (75.0%)	7 (6.0%)	5 (4.3%)	6 (5.2%)	11 (9.5%)	**1.68**	0.5411	0.4162	0.2186	0.7547
Bulimia	Item 2	110 (94.8%)	4 (3.5%)	2 (1.7%)	0.0%	0.0%	1.07	0.6190	0.5076	0.2095	0.7446
Bulimia	Item 3	114 (98.3%)	2 (1.7%)	0.0%	0.0%	0.0%	1.02	0.5205	0.3924	0.2210	0.7573
Bulimia	Item 4	108 (93.1%)	6 (5.2%)	0.0%	1 (0.9%)	1 (0.9%)	1.11	0.4942	0.3623	0.2241	0.7605
Anorexia	Item 5	87 (75.0%)	14 (12.1%)	9 (7.8%)	3 (2.6%)	3 (2.6%)	**1.46**	0.5205	0.3924	0.2210	0.7573
Bulimia	Item 6	112 (96.6%)	4 (3.5%)	0.0%	0.0%	0.0%	1.03	0.3979	0.2547	0.2353	0.7719
Binge-eating	Item 7	95 (81.9%)	11 (9.5%)	3 (2.6%)	4 (3.5%)	3 (2.6%)	1.35	0.5774	0.4585	0.2144	0.7501
BE/Bulimia	Item 8	101 (87.1%)	9 (7.8%)	5 (4.3%)	1 (0.9%)	0.0%	1.19	0.5661	0.4452	0.2157	0.7515
BE/Anorexia	Item 9	96 (82.8%)	12 (10.3%)	4 (3.5%)	0.0%	4 (3.5%)	1.31	0.6710	0.5703	0.2035	0.7375
Anorexia	Item 10	82 (70.7%)	20 (17.2%)	5 (4.3%)	4 (3.5%)	5 (4.3%)	**1.53**	0.5854	0.4678	0.2134	0.7491
Anorexia	Item 11	76 (65.5%)	19 (16.4%)	3 (2.6%)	12 (10.3%)	6 (5.2%)	**1.73**	0.3990	0.2559	0.2351	0.7718
Anorexia	Item 12	66 (56.9%)	25 (21.6%)	10 (8.6%)	11 (9.5%)	4 (3.5%)	**1.81**	0.5088	0.3790	0.2224	0.7588
Mean total score							16.3			Overall correlation 0.2195	Overall alpha **0.7714**
Mean total score per item							1.36				

Total score- total of scores for 12 items; Mean total score- mean total for the Cohort; Mean total score per item = total score/12; Mean scores in bold represent item means higher than overall mean total per item; PEBS- Prenatal Eating Behaviours Screening Tool; Overall Cronbach's alpha = 0.77 BE- Binge-eating; ED- Eating disorders; Items assigned to ED diagnosis based on expert opinion as mentioned in Claydon et.al

### Validity and reliability of PEBS-Arabic

The first round of content validity testing of version 1 of the translated tool revealed that items 1, 2, 6, 7, and 8 had ICVI scores of 0.40–0.80, which indicated poor content validity. Thus, these items were revised as with version 2 until the second round of CVI testing indicated both ICVI and SCI scores of 1.00 which passed the content validity test.

For construct validity, confirmatory factor analysis was performed using a polychromic matrix and a single factor loading, as shown in Table 3. Most items (10 out of 12) loaded with an acceptable correlation coefficient ≥ 0.65, with items 11 and 12 having loadings > 0.50 after varimax rotation. Most of the items had a retained uniqueness of < 50%. The pattern and magnitude of the loadings are similar to those of the validation sample used by Claydon et al. in the questionnaire development study, which had an acceptable model fit [14]. In our study, the single-factor solution accounted for only 51% of the variability (AIC = 497.09).

**Table 3:** Confirmatory and exploratory factor analysis using a polychoric correlation matrix of PEBS items, with varimax rotation

ED screened	PEBS items	Single factor solution		Two-factor solution			Three-factor solution			
		Factor 1	Uniqueness	Factor 1 (Bulimia)	Factor 2 (Anorexia)	Uniqueness	Factor 1 (Bulimia)	Factor 2 (BE)	Factor 3 (Anorexia)	Uniqueness
Bulimia	Item 1	0.6595	0.57	**0.7056**		*0.46*	**0.4237**	0.6329		*0.41*
	Item 2	0.7595	*0.42*	**0.7926**		*0.31*	**0.8738**			*0.16*
	Item 3	0.7119	*0.49*	**0.9706**		*0.06*	**0.8129**			*0.05*
	Item 4	0.7411	*0.45*	**0.7261**		*0.38*	**0.3290**	0.8044		*0.21*
	Item 6	0.6891	0.53	**0.6249**		0.50	**0.7513**			*0.34*
Binge-eating	Item 7	0.7540	*0.43*		0.7299	*0.34*		**0.4455**	0.6682	*0.31*
BE/Bulimia	Item 8	0.7566	*0.43*	**0.5414**		*0.43*	0.7660			*0.17*
BE/Anorexia	Item 9	0.8961	*0.20*		**0.6408**	*0.19*		**0.5473**	0.5508	*0.18*
Anorexia	Item 5	0.6487	0.58		**0.8440**	*0.28*			**0.8044**	*0.22*
	Item 10	0.7217	*0.48*		**0.8762**	*0.20*			**0.8500**	*0.20*
	Item 11	0.5063	0.51		**0.7637**	*0.42*			**0.7751**	*0.34*
	Item 12	0.6241	0.62	0.5307	**0.3440**	0.60		0.8151		*0.27*
Eigenvalues	6.07		4.13	3.72		3.22	3.19	2.71		
Cumulative variation accounted for by factors	0.51		0.34	0.65		0.27	0.53	0.76		
AIC	497.09		497.09	424.26		497.09	424.26	305.97		

Numbers in bold represent highest correlations between factor and item more than 0.3 with expected loading; Numbers in red represent cross-loading into other factors; Uniqueness represents the remaining variation in the item not explained by the factor- < 50% are in italics; ED- eating disorder; BE- Binge-eating

Additional exploratory factor analyses (EFA) were done using two-factor and three-factor solutions. In the two-factor solution, items 1–6 and 8 loaded well into one factor, with most loadings above 0.7. These items were intended to screen for bulimia. Similarly, items 5 and 9–11 load well into the second factor, intended to screen for anorexia. Item 7 was intended for BE but loaded strongly into the second factor, and item 12, intended for anorexia, loaded into the first factor. The two-factor solution accounted for 65% of variability with a better fit than the single factor (AIC = 424.26). The best fit for the data was the 3-factor solution (76% variability, AIC = 305.97). However, there were multiple cross-loadings for items 1, 4, and 7–9, with bulimia retaining items 2,3 and 6 and anorexia retaining items 5, 10, and 11. Items 7, 8, and 9 intended for BE did not load together in one factor. Considering this, the two-factor solution works better than the other options in our population, with all items intended for bulimia and most items intended for anorexia correlating strongly with the respective factors (Table 3).

The overall Cronbach's alpha for the questionnaire, including all 12 items, was 0.77 (error variance of 0.41), representing a good and acceptable internal consistency or reliability. The alpha remained approximately the same when excluding any of the items, as shown in Table 2. The overall inter-item correlation was acceptable at 0.22, implying that the items are distinct from each other but represent the same overall construct. It did not drop below 0.2 when removing any of the items.

## Discussion

### Main findings

This study performed an Arabic translation of the PEBS tool (PEBS-Arabic) and piloted it for the first time in pregnant women. The psychometric analysis of the translated and adapted questionnaire revealed excellent content validity and good reliability. The confirmatory factor analysis demonstrated acceptable loadings in the recommended three-factor solution. Assigning terms in Arabic matching those in English and reflecting gender distinction using feminine forms were the highlighted changes but were reviewed and tested to ensure that the Arabic version constantly capture the original construct. Additionally, this study provides an overview of the prenatal eating behaviors of a cohort of Arab women in Qatar and the correlation between eating behaviors and the demographic profile. To date, no other studies have translated PEBS into other languages. This original article is the first study to assess the validity and reliability of an Arabic version.

### Interpretation of results

Previous studies report the associations between altered eating behaviors among pregnant mothers and adverse outcomes such as growth restriction and preterm birth in those with restrictive ED. Data from Norway suggests that BE among mothers is associated with high birth weight and large-for-gestational-age babies [10]. Furthermore, BE can result in increased maternal weight gain during pregnancy [20], compared to mothers without BE, and is also associated with high sugar and fat intake [21]. During the postpartum stage, mothers with a clinically significant ED find it challenging to balance the desire to restrict caloric intake and the desire to eat [22]. Contrasting these reports, a large population-based study from the Netherlands reported that, although breastfeeding initiation is less with ED, they have a higher diet quality, assessed using a diet score consisting of type of food and quality of nutrient intake, during pregnancy and for their infants compared to women without ED [23]. This finding could be due to the higher score in the diet quality checklists in women with ED who might choose to consume less meat and fat in order to restrict further weight gain, or who are more aware of healthier options and have a desire to improve their diet during pregnancy.

There were several determinants of eating behavioral problems during pregnancy. Chang et al. showed that maternal employment resulting in a lack of time to plan and cook balanced meals causes pregnant women to increase fast food consumption and less balanced dietary choices [24]. Increased awareness and education of healthcare providers can make positive changes and motivate pregnant mothers to make healthier balanced dietary choices [25]. Additionally, socioeconomic status, parity, and education were found to be associated with balanced dietary habits among pregnant mothers in Nigeria [26]. In our cohort, higher education seems to be associated with higher scores which are probably due to an increased exposure to peer pressure and misconception of ideal body image. Previous study shows that women experience pressure exerted by society and preoccupations about body image to pregnancy [27]. Higher parity seems to be associated with lower scores in our cohort, probably due to the responsibility of managing larger households, as is common in the area, making them less likely to stress about their own body image or weight.

Many symptoms of ED overlap with those of pregnancy, such as nausea, vomiting, bloating, and feeling of fullness, highlighting the importance of using pregnancy-specific instruments such as PEBS [28]. Additionally, a review by Diego-Cordero et al. concluded that cultural beliefs, such as food taboos or restrictions due to fear of inducing pregnancy complications, are strongly associated with pregnant women's food patterns and eating habits [28]. Hence, studying factors for eating behavioral problems should be done in different populations as the experience may differ according to geographical areas. In the Middle East and North Africa region, limited studies assessing prenatal eating disorders were found after comprehensively reviewing three online databases, including grey literature. Several studies examined nutritional deficiencies and eating habits, but none focused on screening for ED during pregnancy. However, an Arabic version of the Disordered Eating Attitudes in Pregnancy Scales or A-DEAPS, which measures the women's attitudes toward eating during pregnancy, has been translated and successfully tested as reliable among Lebanese women [15]. The study revealed that groups of women were at risk, whereas others were already manifesting disordered attitudes toward eating during pregnancy [15]. DEAPS has 2 factors- body image and disordered eating attitudes and, unlike PEBS, does not factor according to specific ED diagnoses. Hence, our study provides an alternative tool in Arabic that can be used to understand ED in pregnancy.

In this study, the content validity was calculated to ascertain that the items in PEBS-Arabic represent the entire domain of interest: eating behavior during the prenatal period. The PEBS-Arabic showed performed better than recommended values [17], which indicates that the translated items are relevant and represent entirely the domain of interest. The CFA and EFA were performed to examine if the translated items still measured their intended construct. The pattern and magnitude of the loadings of the single-factor solution are similar to findings in the validation sample used by Claydon et.al. In the questionnaire development study. We additionally tested a 2-factor solution, and the items loaded well into bulimia and anorexia, with better correlation coefficients than the single-factor. The 3-factor solution, including BE, had multiple cross-loadings and did not seem to be the best fit for our cohort. Larger studies exploring item correlations with specific ED diagnoses in our population are required to confirm these findings.

In the study by Claydon et al. [14], the PEBS English with the same 12 items was proven highly reliable, with Cronbach’s alpha of 0.95 in the development sample and 0.91 in the validation sample. With PEBS-Arabic, the test indicated good and acceptable reliability with Cronbach’s α of 0.77, which is expected with repeat reliability testing, especially in a different geographical and clinical setting, with a smaller sample size.

### Strengths, limitations and research implications

This is the first non-English translation of PEBS and one of the few studies exploring ED in pregnancy from the Middle East. Performing a psychometric analysis of the translated version will help in the incorporation of a validated PEBS-Arabic tool in routine antenatal care in Qatar, as well as promote use in other Arabic-speaking countries. The sample size was adequate to test reliability at approximately 10 women per item in the questionnaire. Developing a self-administered questionnaire in the local language is essential to understanding ED in pregnancy, especially when there is an associated social stigma. Larger prospective studies using PEBS-Arabic to evaluate antenatal scores can help understand the clinical condition better and help improve risk assessment, ultimately improving maternal, fetal and child health.

Despite the acceptable validity and reliability of the translated questionnaire, some limitations need to be kept in mind while interpreting the clinical correlations. The study was a cross-sectional review of women admitted in the antenatal units of the tertiary centre in their third trimesters, and as such, the correlations might not be generalizable to all pregnant women in the country. Additionally, there is a possibility that women who answered the questionnaire displayed more health-conscious behaviors compared to those who chose not to participate, which can further impact generalizability. However, the main aim here was to translate and test the questionnaire. Further larger studies, including other hospitals, are required to improve the generalizability of the findings and to determine associations with maternal demographics and with specific ED diagnoses. The psychometric property findings of PEBS-Arabic can be further supported by other tests such as divergent, convergent, and other validity tests and need to be evaluated further according to trimesters of pregnancy, as dietary habits tend to change between trimesters.

## Conclusion

In conclusion, the PEBS-Arabic has excellent content validity and good construct validity and reliability. Maternal education level and the number of previous children showed a direct association with their prenatal eating behaviors. Therefore, PEBS-Arabic has shown promise in assessing and identifying eating behaviors and potential eating disorders in pregnancy. This tool is valuable in ensuring early detection and treatment of ED, especially for those with limited capacity to understand the items of the original version of the tool due to the English language. The study is also valuable among healthcare clinicians working in the Arab region as it will enhance awareness among healthcare workers regarding these less-understood clinical presentations and ultimately improve maternal motivation for a balanced dietary style.

## Data Availability

The datasets generated and/or analysed during the current study are not publicly available due to confidentiality and hospital policies but are available from the corresponding author upon reasonable request**.**

## Abbreviations

AIC: Akaike information criteria
BE: Binge eating
CFA: Confirmatory factor analysis
CVI: Content validity index
DEBS: Disordered eating behavior scale
DEAPS: Disordered eating attitudes in pregnancy scales
ED: Eating disorders
EDE-Q: Eating disorder examination questionnaire
EDI-2: Eating disorder inventory-2
EFA: Exploratory factor analysis
I-CVI: Item content validity index
IQR: Interquartile range
MRC: Medical research centre
MSA: Modern standard Arabic
PEBS: Prenatal eating behavior screening tool
S-CVI: Scale content validity index
SD: Standard deviation
WWRC: Women’s wellness and research centre
